# Cancer prevention with rapamycin

**DOI:** 10.18632/oncotarget.28410

**Published:** 2023-04-14

**Authors:** Mikhail V. Blagosklonny

**Affiliations:** ^1^Roswell Park Comprehensive Cancer Center, Buffalo, NY 14263, USA

**Keywords:** chemoprevention, lung, rapamycin, aging, cancer

## Abstract

Rapamycin (sirolimus) and other rapalogs (everolimus) are anti-cancer and anti-aging drugs, which delay cancer by directly targeting pre-cancerous cells and, indirectly, by slowing down organism aging. Cancer is an age-related disease and, figuratively, by slowing down time (and aging), rapamycin may delay cancer. In several dozen murine models, rapamycin robustly and reproducibly prevents cancer. Rapamycin slows cell proliferation and tumor progression, thus delaying the onset of cancer in carcinogen-treated, genetically cancer-prone and normal mice. Data on the use of rapamycin and everolimus in organ-transplant patients are consistent with their cancer-preventive effects. Treatment with rapamycin was proposed to prevent lung cancer in smokers and former smokers. Clinical trials in high-risk populations are warranted.

## INTRODUCTION

The mTOR (Target of Rapamycin) pathway is involved in both cancer and aging. Furthermore, common cancers are age-related diseases, and their incidence increases exponentially with age. Rapamycin (sirolimus) and other rapalogs (temsirolimus, everolimus) may delay cancer by targeting directly pre-cancerous cells and by slowing down organism aging.

### Rapamycin delays tobacco-related and lung cancer in mice

In 2007, it was demonstrated that rapamycin prevents lung cancer in mice caused by the tobacco-specific carcinogen NNK [1]. Mice were treated with NNK at the age of 6 weeks. In one of experiments, treatment with rapamycin (every-other-day) was started one week after NNK exposure. Rapamycin decreased tumor multiplicity by 90%. Phenotypic progression of tumors was slowed, tumors were smaller in size by 74% due to decreased cell proliferation. Granville et al. envisioned a clinical trial of rapamycin for smokers at high risk of lung cancer [1].

Supporting these results, Patlolla et al. showed that rapamycin delayed (or prevented) development of NNK-induced lung adenoma and progression from lung adenoma to adenocarcinoma in mice [2]. When treatment with rapamycin was started early (three weeks after NNK), rapamycin suppressed development of lung adenoma and adenocarcinoma [2]. For late intervention (rapamycin treatment was started 20 weeks after NNK exposure) rapamycin suppressed progression from lung adenoma to lung cancer. The authors concluded that rapamycin is effective even after dysplastic adenoma or early adenocarcinoma stages and may be useful for high-risk lung cancer people [2].

Yan et al. found that treatment with rapamycin for 14 weeks, beginning 12 weeks after administration of a polycyclic aromatic hydrocarbon [benzo(*a*)pyrene] (BP), decreased lung tumor load by 84% [3].

In a model of EGFR mutant lung cancer in mice, rapamycin prevented or slowed down tumor development. Median overall survival was prolonged by more than three-fold [4].

In a different mouse model, 4-nitroquinoline-1 oxide (a surrogate of tobacco exposure) caused head and neck squamous cell carcinoma (HNSCC). This tobacco-mimicking carcinogen leads to the appearance of preneoplastic and tumoral lesions, with 100% incidence. Many of these lesions progressed into highly malignant squamous cell carcinomas few weeks after carcinogen withdrawal. The Akt-mTOR was overactivated as an early event in dysplastic lesions. Rapamycin delayed the onset and slowed progression of tumorigenesis [5]. Chronic administration of rapamycin prevented the malignant conversion of precancerous lesions [5]. It was suggested to use rapamycin for chemoprevention of upper aerodigestive tract cancers [5, 6].

### Rapamycin prevents cancer and extends lifespan in cancer-prone mice

In transgenic HER-2/neu cancer-prone mice, rapamycin treatment (started 2 months after birth) decreased rate of aging, increased lifespan, and suppressed carcinogenesis. Rapamycin delayed tumor onset, decreased the number of tumors per animal and tumor size, increasing maximal lifespan by 12.4% [7]. In the follow-up work in these mice, some degree of cancer prevention can be achieved by low doses of rapamycin [8].

In highly tumor-prone p53−/− mice, rapamycin extended the mean lifespan by 30% and delayed tumor development [9].

In heterozygous p53+/− mice, rapamycin also extended the mean lifespan when treatment started early in life and decreased the incidence of tumors [10].

In *Rb1*^+/−^ mice, rapamycin extended lifespan and delayed the onset and/or progression of neuroendocrine tumors [11].

In cancer-prone germline PTEN mutant mice, long-term treatment (started at the age of 6 week) with low doses of rapamycin extended lifespan and delayed tumor development [12].

In male mice with prostate epithelium-specific *Pten*-knockout mouse prostate cancer model, low dose of rapamycin (formulated as Rapatar) was effective in suppressing proliferation of prostate epithelial cells and prevention of prostate cancer. A higher dose activated feedback circuits that decreased the drug’s tumor preventive efficacy [13].

Deletions of transforming growth factor-β receptor I and PTEN in oral mucosa resulted in spontaneous development of HNSCC with 100% penetrance. Rapamycin treatment delayed progression of papilloma and the onset of squamous cell carcinoma in the head and neck region as well as the oral cavity and increased life span and median survival almost two-fold [14].

Apc(Min/+) mice exhibit multiple intestinal neoplasia (MIN), which causes death by 6 months. Short-term treatment with everolimus and rapamycin reduced the number of polyps and their size [15, 16]. Importantly, chronic rapamycin improved survival of Apc(Min/+) mice in a dose-dependent manner [17]. A high dose of enterically targeted rapamycin (eRapa) extended the median lifespan beyond normal median lifespan of wild-type syngeneic mice. Based on these results it was suggested that rapamycin may be effective for cancer prevention in people with familial adenomatous polyposis [17, 18].

Everolimus (RAD001) delays tumor onset and progression in a transgenic mouse model of ovarian cancer [19]. Tumor burden was decreased by 84%. Approximately 30% of everolimus-treated mice developed early ovarian carcinoma confined within the ovary, whereas all placebo-treated mice developed advanced ovarian carcinoma [19]. The authors suggested rapamycin for women at high familial risk of ovarian cancer [19].

### Rapamycin delays cancer in normal mice

In numerous studies, rapamycin extended lifespan in normal strains and genetically heterogeneous mice and wild mice (see for references [20]). Cancers are the leading cause of death in most mouse strains used for these studies [21, 22]. Presumably, when death was delayed, then the cause of death (mostly cancer) was delayed too. Some studies investigated this assumption specifically.

In genetically heterogeneous mice, administration of rapamycin started at the age of 9 and 20 months extended lifespan and delayed cancer [21, 22]. It was even speculated that “longevity extension in these mice might reflect inhibition of multiple forms of neoplastic disease” [23]. Alternatively, it was suggested that, by slowing and delaying aging, rapamycin delayed cancer [23, 24]. Similarly, lifelong administration of rapamycin (starting from age of 2 months) increased lifespan and delayed spontaneous cancer in mice [24]. Neff et al. detected several cancers in aged mice, including lymphoma, hepatocellular carcinoma, histiocytic sarcoma, and bronchoalveolar adenocarcinoma, as well as precancerous lesions in male C57BL/6J mice [25]. Rapamycin significantly reduced the proportion of aged mice presenting with cancers and/or precancerous lesions in the 16-month cohort (control, 4 of 10; rapamycin, 0 of 15), but not at the older ages of 25-month and 34-month. The authors concluded that rapamycin delayed cancer and cancer-caused death in male C57BL/6J mice [25].

### Cancer prevention in humans

Solid organ (kidney, liver, lung, heart) transplantation is associated with increased risk of cancer and especially of non-melanoma skin cancer.

Starting from 2004, numerous studies demonstrated that rapamycin and everolimus reduced the incidence of various cancers in organ transplant patients [26–34].

For example, Mathew et al. showed that Sirolimus (rapamycin) protected renal transplant patients from skin cancer even when given in combination with CsA (CsA increases incidence of skin cancer) [26].

Kauffman et al. also demonstrated that sirolimus and everolimus treatment is associated with a significantly decreased risk of any *de novo* malignancy and non-skin solid malignancy [27].

Piselli et al. also found that use of mTOR inhibitors significantly reduced the risk (by 46%) of all cancers combined [30].

mTOR inhibition was associated with a reduced risk of basal cell carcinoma of the skin after kidney transplantation [31]. Rapamycin (sirolimus) treatment was associated with decreased incidence of lymphoproliferative disorder after heart transplantation [34].

### Rapamycin and its analogs for cancer therapy

Rapamycin analogs (temsirolimus and everolimus) are approved for various cancers: renal, breast, lung and others.

On May 30, 2007, temsirolimus (Torisel) was approved for the treatment of advanced renal cell carcinoma (RCC) [35, 36]. In comparison with treatment with interferon-a (the best treatment in 2007), temsirolimus further increased overall survival in patients with metastatic renal-cell carcinoma. Median overall survival in the interferon group, the temsirolimus group, and the combination-therapy group were 7.3, 10.9, and 8.4 months, respectively. There was also a statistically significant longer progression-free survival (PFS) time for the temsirolimus arm than for the IFN-alpha: 5.5 months versus 3.1 months [36].

On Mar 30, 2009, everolimus (rapamycin analog) was approved by the FDA as a first treatment for patients with advanced kidney cancer after failure of either sunitinib or sorafenib. Treatment with everolimus prolonged progression-free survival relative to placebo in patients with metastatic renal cell carcinoma that had progressed on other targeted therapies. Median progression-free survival was 4.0 vs. 1.9 months [37].

On July 20, 2012, everolimus was approved by the FDA for use in combination with exemestane to treat women with advanced hormone receptor-positive, HER2-negative breast cancer [38]. At the interim analysis, median progression-free survival was 6.9 months with everolimus plus exemestane and 2.8 months with placebo plus exemestane. Median progression-free survival was 10.6 months and 4.1 months, respectively, according to central assessment. Everolimus combined with an aromatase inhibitor improved progression-free survival in patients with hormone receptor-positive advanced breast cancer previously treated with nonsteroidal aromatase inhibitors [38].

On February 26, 2016, the FDA approved everolimus (Afinitor) for the treatment of adult patients with progressive, well-differentiated, non-functional neuroendocrine tumors (NET) of gastrointestinal or lung origin with unresectable, locally advanced or metastatic disease. Median PFS were 11 months and 3.9 months in the everolimus and placebo arms [39].

### Delaying (prevention) vs. treating cancer

To be a highly effective cancer-preventive drug, rapamycin does not need to cure cancer or even be effective in treating advanced, heterogeneous and metastatic tumors, harboring numerous oncogenic mutations and failed previous therapy. To prevent cancer, rapamycin does not need to kill cancer cells (rapamycin does not kill cells) or stop tumor progression (it merely slows it down).

**Figure 1:**
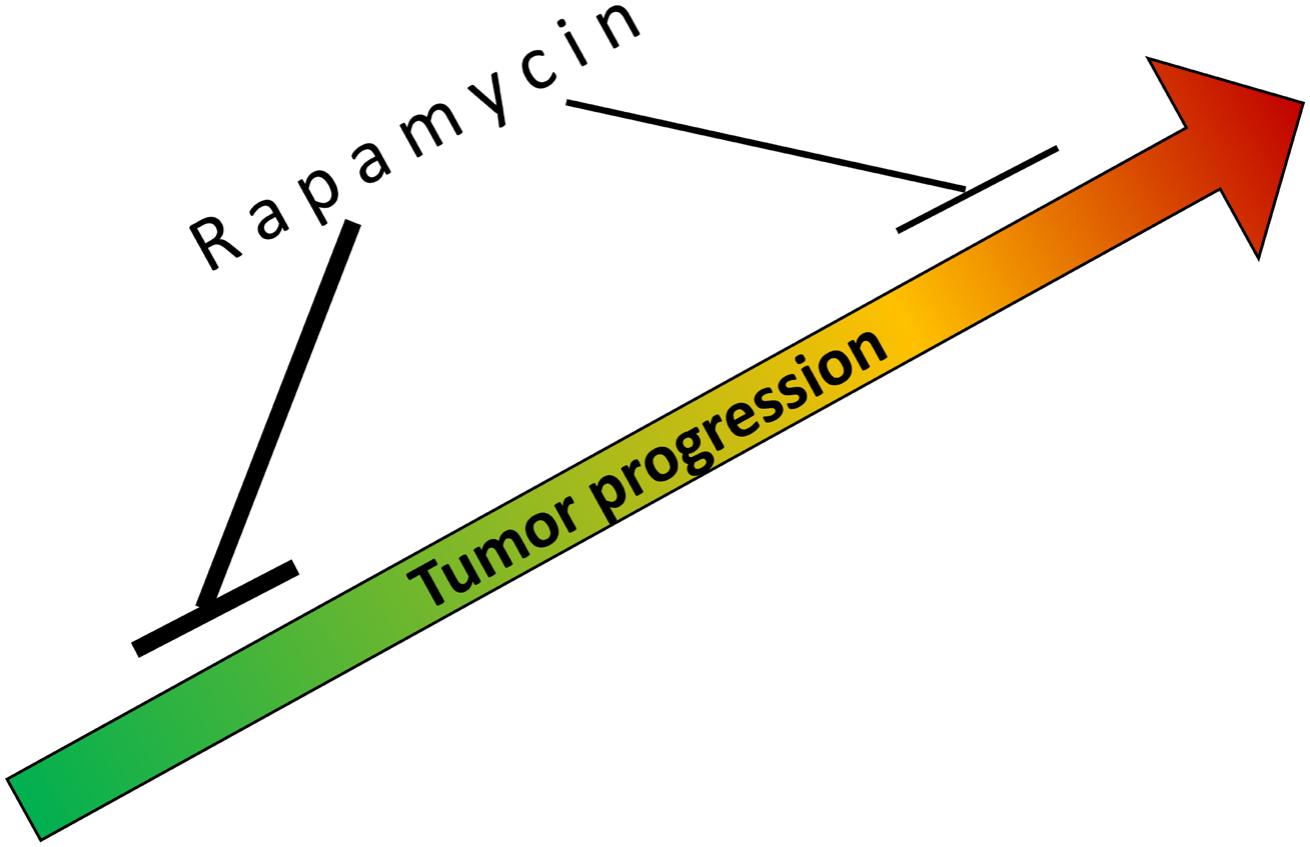
Rapamycin slows tumorigenesis, tumor progression and growth. Rapamycin is more potent at early stage of tumorigenesis (green color) than in advanced and pre-treated cancer (red color).

As we discussed earlier, rapamycin slows cell proliferation and tumor progression, thus delaying the onset of cancer in tobacco-carcinogen-treated mice, in both genetically cancer-prone and normal mice (Figure 1). (In cell culture, rapamycin slows cell proliferation 2–10 fold and slows geroconversion to cell senescence 3-fold [40]). Rapamycin figuratively slows down time [41].

For cancer prevention, treatment with rapamycin should last many years (in humans). In numerous studies, mice were treated for almost a lifetime. It was not only well-tolerated but also improved healthspan and lifespan (see for references [42, 43]).

**Figure 2:**
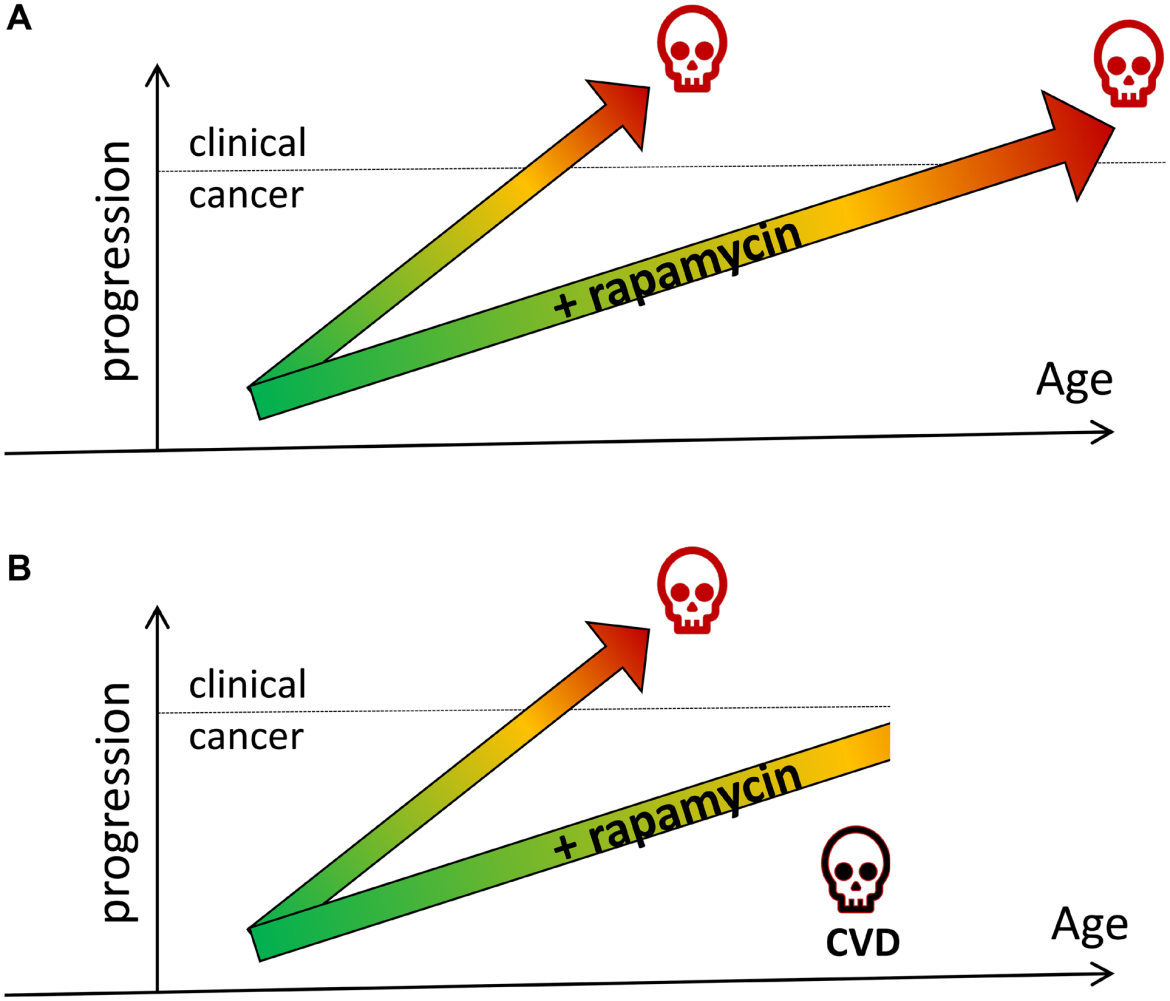
Rapamycin prevents cancer by slowing tumor progression (hypothetical schema). (**A**) Rapamycin slows tumor progression and delays cancer and death from cancer. (**B**) Rapamycin slows tumor progression and delays cancer. A person dies from another cause (e.g., cardiovascular disease, CVD) before cancer developed.

If, hypothetically, in humans, a low dose of rapamycin would slow pre-cancer cell proliferation and tumor progression just 2-fold, and a person would be treated for 40 years, then the onset of cancer would be delayed for 20 years. Then this person may die later in life from another age-related disease, for example CVD. Such a significant cancer delay can be viewed as cancer prevention (Figure 2).

In humans, lung cancer may take 20 years to develop, with smoking driving mutations, even 20 years after quitting [44].

In tobacco-carcinogen-induced lung tumorigenesis in mice, rapamycin delays tumors by slowing down tumor progression and cell proliferation. When treatment is started early, rapamycin decreases not only the size of tumors and tumor burden, but also the number of tumors per animal. When treatment is started later (after tumors develop), rapamycin does not decrease tumor multiplicity but slows tumor progression and growth, making them smaller [1–3].

Using a two-stage skin carcinogenesis protocol with DMBA (carcinogen) and TPA (tumor promoter), rapamycin (given topically 30 minutes prior to TPA) exerted a powerful anti-promoting effect, reducing both tumor incidence and tumor multiplicity [45]. Furthermore, rapamycin abolished tumor development when administered prior to both DMBA and TPA [46].

Rapamycin protects HPV-E6/E7 expressing tissues from developing of squamous cell carcinoma [46].

It was concluded that rapamycin is a potent chemopreventive agent [47, 48]. “Rapamycin suspends progression of low-grade cancers, preventing invasive conversion of *in situ* malignancy, or delaying malignant transformation of established pre-malignant conditions” [48].

### Rapamycin slows aging, thus delaying cancer further

Rapamycin can delay cancer not only by targeting precancerous/cancerous cells directly, but also by slowing down organismal aging [49]. It is theoretically predictable that rapamycin delays age-related diseases in part by slowing aging [50]. Certainly, rapamycin extends lifespan in other ways beyond preventing cancer.

1. Rapamycin delays numerous age-related diseases other than cancer. For example, rapamycin (sirolimus) or its analog (everolimus) attenuate atherosclerosis in rabbits [51], mice [52] and humans [53]. Thus, a prospective randomized trial showed that rapamycin (sirolimus) decreased carotid atherosclerosis in organ-transplant patients [53].
2. Rapamycin extends lifespan in species that do not have cancer: the *C. elegans* worm [54], the freshwater cnidarian Hydra [55], and *Daphnia magna* [56]. Rapamycin also extends the lifespan of yeast [57].
3. A brief treatment with rapamycin very early in life extends lifespan in mice [56, 58, 59]. There are no pre-cancer cells so early in life, and the treatment with rapamycin is brief. One explanation is that by re-programming development-driven aging, rapamycin retards aging and therefore delays cancer [20].
4. Rapamycin slows geroconversion (acquisition of the senescent phenotype) in mammalian cells. First, geroconversion may be linked to organism aging [60]. Second, senescent stroma stimulates tumor growth [61–63]. Reversing the aging stromal phenotype with rapamycin prevents carcinoma initiation [61], and rapamycin suppressed the ability of senescent fibroblasts to stimulate tumor growth in mice [64, 65].

## CONCLUSION

In several dozen murine models, rapamycin robustly and reproducibly delays cancer and, in some cases, prevents cancer over a lifetime. It was repeatably proposed that clinical trials in high-risk populations are warranted. A decade-long treatment with rapamycin may be employed to prevent lung cancer in smokers and former smokers. However, decades-long trails are unlikely to be started in the near future. Accidental data on the use of rapamycin (Sirolimus) and everolimus in organ-transplant patients is consistent with their cancer-preventive effects. However, in these patients, their use in combination with other immunosuppressants makes interpretations difficult.

**Figure 3:**
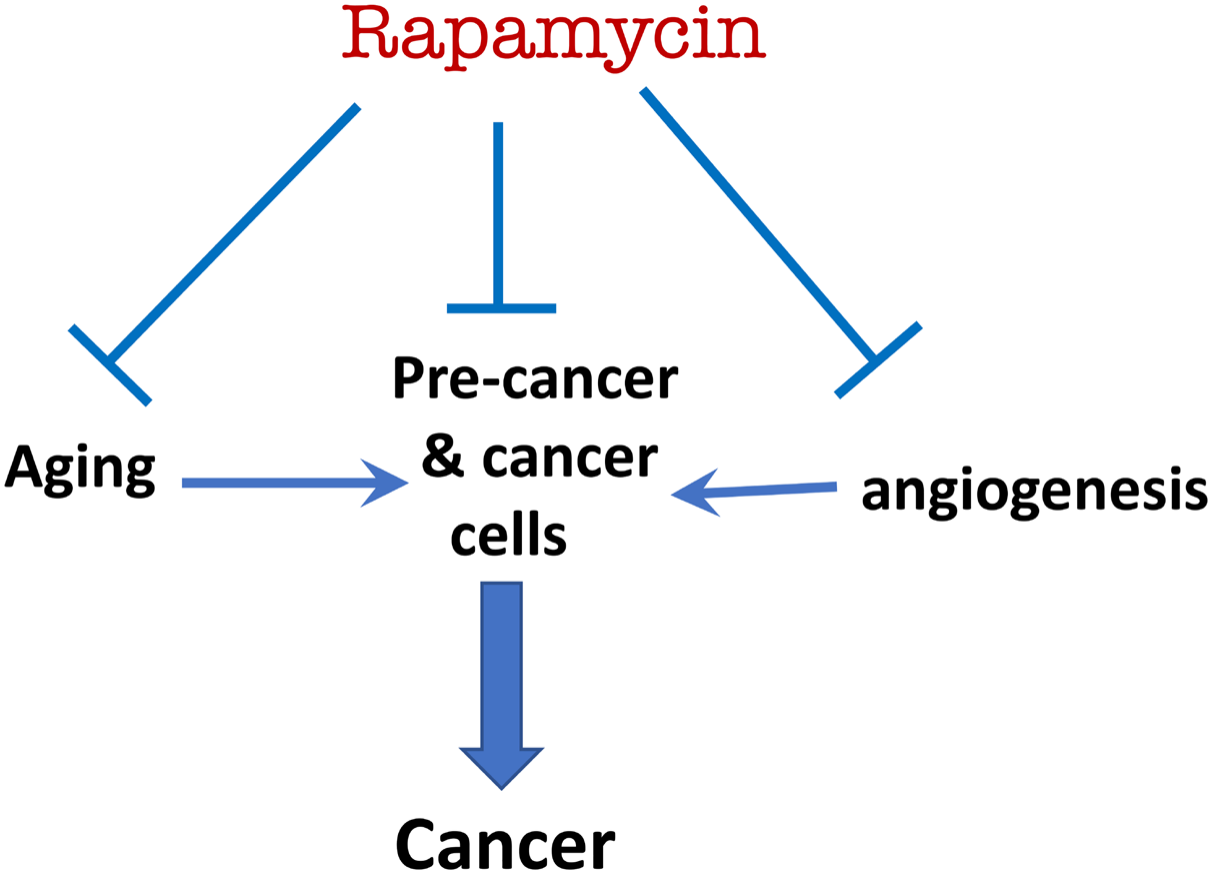
Rapamycin prevents cancer by direct (suppressing pre-cancerous/cancer cell) and indirect mechanisms (suppressing aging and angiogenesis).

The experience of treatment of cancer patients with mTOR inhibitors is also in agreement with their cancer-preventive effects. Although rapalogs do not cure cancer and infrequently cause remission, they can slow down progression even in advanced tumors, and this activity is sufficient for cancer prevention. Also, long-term treatment with rapamycin slows down aging, a major risk factor for cancer (Figure 3). Notably, delaying cancer is form of cancer prevention. Consider a scenario: rapamycin delays cancer for 2 years, during which this person dies from myocardial infarction (Figure 2). In this case postponing cancer is cancer prevention.

Currently, an increasing number of healthy people use rapamycin off-label to slow down aging. Perhaps in ten or twenty years from now, data will accumulate for retrospective analysis of cancer-prevention with rapamycin in humans.
